# A new amino acid substitution (Ala-205-Phe) in acetolactate synthase (ALS) confers broad spectrum resistance to ALS-inhibiting herbicides

**DOI:** 10.1007/s00425-015-2399-9

**Published:** 2015-09-09

**Authors:** James T. Brosnan, Jose J. Vargas, Gregory K. Breeden, Logan Grier, Raphael A. Aponte, Stefan Tresch, Martin Laforest

**Affiliations:** Plant Sciences Department, The University of Tennessee, 2431 Joe Johnson Drive, Knoxville, TN 37996 USA; BASF Corporation, 26 Davis Dr., Research Triangle Park, NC 27709 USA; BASF SE, Speyerer Str. 2, 67117 Limburgerhof, Germany; Horticulture R&D Center, Agriculture and Agri-Food Canada, St-Jean-sur-Richelieu, QC Canada

**Keywords:** Acetolactate synthase, Annual bluegrass, Phenylalanine, Turf, Weed resistance

## Abstract

**This is a first report of an Ala-205-Phe substitution in acetolactate synthase conferring resistance to imidazolinone, sulfonylurea, triazolopyrimidines, sulfonylamino-carbonyl-triazolinones, and pyrimidinyl (thio) benzoate herbicides.**

Resistance to acetolactate synthase (ALS) and photosystem II inhibiting herbicides was confirmed in a population of allotetraploid annual bluegrass (*Poa annua* L.; POAAN-R3) selected from golf course turf in Tennessee. Genetic sequencing revealed that seven of eight POAAN-R3 plants had a point mutation in the *psbA* gene resulting in a known Ser-264-Gly substitution on the D1 protein. Whole plant testing confirmed that this substitution conferred resistance to simazine in POAAN-R3. Two homeologous forms of the ALS gene (ALSa and ALSb) were detected and expressed in all POAAN-R3 plants sequenced. The seven plants possessing the Ser-264-Gly mutation conferring resistance to simazine also had a homozygous Ala-205-Phe substitution on ALSb, caused by two nucleic acid substitutions in one codon. In vitro ALS activity assays with recombinant protein and whole plant testing confirmed that this Ala-205-Phe substitution conferred resistance to imidazolinone, sulfonylurea, triazolopyrimidines, sulfonylamino-carbonyl- triazolinones, and pyrimidinyl (thio) benzoate herbicides. This is the first report of Ala-205-Phe mutation conferring wide spectrum resistance to ALS inhibiting herbicides.

**Electronic supplementary material:**

The online version of this article (doi:10.1007/s00425-015-2399-9) contains supplementary material, which is available to authorized users.

## Introduction

Annual bluegrass (*Poa annua* L.) is a problematic weed of both warm- and cool-season turf that grows on every continent including Antarctica (Scott 1987). An allotetraploid species, annual bluegrass is theorized to be the product of hybridization between *Poa supina* Schrad. and *Poa infirma* Kuth. (Mao and Huff 2012). Chromosome doubling has been thought to affect the likelihood of herbicide resistance developing in annual bluegrass (McElroy et al. 2013). There are more instances of annual bluegrass developing herbicide resistance in managed turf than any other weed species (Heap 2014). This may be related to both high fecundity and intense herbicide use as Gressel and Levy (2006) explained that repeated herbicide application on highly prolific annual weeds with short life cycles exposes a large number of individuals to selection pressure for resistance alleles. It has been estimated that annual bluegrass can accumulate 185,000 viable seeds m^−2^ in the top 2.5 cm of soil (Watschke et al. 1979). Moreover, herbicidal inhibitors of acetolactate synthase (ALS), microtubule assembly, photosystem II (PSII), and 5-enolpyruvylshikimate-3-phosphate synthase are often applied for annual bluegrass control in turf without rotating among different herbicidal mechanisms of action.

Globally, annual bluegrass populations have developed resistance to nine different herbicidal mechanisms of action; however, instances of a single population developing multiple resistance are limited (Heap 2014). A population of annual bluegrass resistant to ALS and PSII inhibiting herbicides (POAAN-R3) was identified on a golf course in Memphis, Tennessee (Brosnan et al. 2015). Trifloxysulfuron applications at rates as high as 223 g ha^−1^ only controlled POAAN-R3 ≤40 %. Similarly, simazine at 140 to 9000 g ha^−1^ only resulted in ≤20 % control. Using methods of Singh et al. (1988), the researchers found no differences in the in vitro activity of ALS in POAAN-R3 and susceptible annual bluegrass plants exposed to increasing foramsulfuron concentrations from 0 to 100 μM, suggesting that non-target mechanisms could explain reduced efficacy of POST herbicide applications.

Point mutations conferring resistance to ALS and PSII inhibiting herbicides are well documented. Amino acid substitutions at positions 122, 197, 205, 376, 377, 574, 653, and 654 on the gene coding for ALS target site [numbered according to corresponding sequence of *Arabidopsis thaliana* (L.) Heynh.] have been identified in weed populations resistant to sulfonylurea and imidazolinone herbicides (Tranel and Wright 2002; Yu and Powles 2014a). In annual bluegrass, a Trp-574-Leu substitution on ALS has been shown to confer a high level of resistance to foramsulfuron, trifloxysulfuron, imazaquin, and bispyribac-sodium (McElroy et al. 2013). Multiple copies of the ALS gene were identified in this annual bluegrass population, both with and without this mutation (McElroy et al. 2013). Low-level (<10 fold) resistance to both sulfonylurea and imidazolinone herbicides has also been reported in common cocklebur (*Xanthium strumarium* L.) with an Ala-205-Val substitution (Woodworth et al. 1996). Annual bluegrass populations resistant to PSII inhibiting herbicides such as simazine and amicarbazone often have a point mutation on the *psbA* gene that codes for a Ser-264-Gly mutation on the D1 protein targeted by PSII inhibiting herbicides (Kelly et al. 1999; Perry et al. 2012). However, five other mutations on the *psbA* gene have been linked with weed resistance to PSII inhibiting herbicides (Beckie and Tardif 2012).

Individual weed populations resistant to ALS and PSII inhibiting herbicides have been shown to have multiple mutations causing herbicide resistance. Single populations of Powell amaranth (*Amaranthus powelli*) in cropping systems have been shown to have both a Ser-264-Gly mutation on *psbA* as well as Thr-653-Ser mutations in ALS resulting in resistance to atrazine and imazethapyr, respectively (Diebold et al. 2003). A population of Italian ryegrass (*Lolium multiflorum*) in Oregon was found to have both a *psbA* Ser-264-Gly mutation and an ALS Trp-574-Leu substitution that conferred resistance to atrazine, diuron, hexazinone, imazapyr, and sulfometuron (Liu et al. 2014). Different individual plants in a single population can have as many as six mutations conferring resistance to ALS inhibiting herbicides, as has been reported in *Kochia scoparia* and rigid ryegrass (*Lolium rigidum* Gaudin) (Warwick et al. 2008; Yu et al. 2008). The presence of multiple resistance alleles has been reported in rigid ryegrass with plants exhibiting varying degrees of zygosity for mutations at positions 197 and 574 on the ALS gene (Kaundun et al. 2012).

Analysis of the genes coding for the target sites of ALS and PSII inhibiting herbicides in POAAN-R3 is needed to further elucidate mechanisms endowing resistance to trifloxysulfuron and simazine in this population (Brosnan et al. 2015). Considering previous reports of weed species having multiple mutations associated with ALS herbicide resistance (Warwick et al. 2008; Yu et al. 2008), we hypothesize that POAAN-R3 will contain multiple target site mutations conferring resistance to ALS and PSII inhibitors.

## Materials and methods

Plant culture was similar to Brosnan et al. (2015). Single plants of POAAN-R3 and a susceptible biotype of annual bluegrass purchased commercially (V&J Seed Farm. Woodstock, IL, USA) were established from seed in 16 cm diameter greenhouse containers filled with potting media (Metro-Mix. Sun Gro Horticultural Products. Agawam, MA, USA) and slow-release fertilizer (19 N–6P_2_O_5_–12 K_2_O) in December 2013. Pots were hand thinned to contain one annual bluegrass plant per pot. Supplemental lighting was used in the greenhouse to ensure a minimum day length of 14 h and a minimum light intensity of 21,500 lumens m^−2^. Irrigation was applied as needed.

## Genetic sequencing

For molecular analysis, 5 g of fresh tissue was harvested from eight resistant and two susceptible annual bluegrass plants in the greenhouse and stored until analysis at −80 °C. For isolation of RNA and cDNA synthesis, leaf tissue of two susceptible (S1–S2) and eight resistant (RP1–RP8) annual bluegrass selections was ground in liquid nitrogen and total RNA was extracted using an Ambion RNAqueous-Midi kit (AM1911; Life Technologies. Grand Island, NY USA) with the Plant RNA Isolation Aid (AM9690; Life Technologies. Grand Island, NY USA). To validate the quality of the extracted RNA, 1 μL of the final product was run on a Bioanalyzer 2100 (Agilent Technologies. Santa Clara, CA USA) using the RNA 6000 Nano kit (Agilent Technologies. Santa Clara, CA USA) using methods of Babu and Gassmann (2014). RNA sequencing libraries were produced using TruSeq RNA Sample preparation kits (V2; RS-122-2001; Illumina Inc. San Diego, CA USA).

Libraries of a susceptible (S1) and resistant sample (RP1) were sequenced with a desktop sequencer (Illumina MiSeq machine; Illumina Inc. San Diego, CA USA) with a 2 × 300 bp paired end run using MiSeq Reagent Kit v3 (MS-102-3001; Illumina Inc. San Diego, CA USA). Libraries of susceptible plant S2 and seven resistant plants (RP2–RP8) were sequenced with one lane Illumina HiSeq2000 with 2 × 100 bp paired end run using TruSeq SBS Kit v3 (FC-401-3001 Illumina Inc. San Diego, CA USA). The sequencing raw data were analyzed with FASTQC quality checker (Babraham Bioinformatics 2014), trimmed using EA-Utils fastq-mcf (https://code.google.com/p/ea-utils/) and further analyzed to remove any Illumina adaptor sequences using CutAdapt coding (http://code.google.com/p/cutadapt/). Sequence reads of the S2 plant were assembled using Trinity (http://trinityrnaseq.sourceforge.net/) for de novo assembly. Sequences of ALS and *psb*A genes were identified using the BLAST algorithm (Altschul et al. 1990). To identify possible ALS or *psb*A gene homeologs, all sequencing reads with a minimum 80 % sequence identity to ALS or *psb*A gene fragments from de novo assembly were identified using BLAT (Kent 2002). These sequence reads were assembled using a CLC bio algorithm (version 4.01; CLC Bio, Boston, MA USA) and resulted in a full length contig of one *psb*A gene isoform and two different ALS homeologs named ALSa (GeneBank accession number KT346395) and ALSb (GeneBank accession number KT346396). To analyze relative expression of the ALSa and ALSb homeologs in our annual bluegrass plants, mapped reads using TopHat2 (Kim et al. 2013) were counted using HTseq count (Anders et al. 2015) and ALSa counts were divided by ALSb counts (Table 1).

**Table 1:** Amino acid substitutions at various positions of interest for two isoforms of the acetolactate synthase gene (ALSa, ALSb) and the *psbA* gene coding for the D1 protein in photosystem II in herbicide resistant (RP1–RP8) and susceptible (S1–S2) annual bluegrass (*Poa annua* L.)

		Amino acid substitutions^a,b^					
		ALSa	ALSb				*psb*A
	ALS expression ratio	574	94	205	310	420	264
Selection	ALSa/ALSb	TGG ⇢ TTG	ACC ⇢ GCC	GCC ⇢ TTC	AGC ⇢ TGC	GAG ⇢ GAT	AGT ⇢ GGT
RP1	1.16	W	A	F	C	E	G
RP2	1.19	W	A	F	C	E	G
RP3	1.17	W	A	F	C	E	G
RP4	1.19	W	A	F	C	E	G
RP5	1.35	W	A	F	C	E	G
RP6	1.18	W/L	A	A	C	E/D	S
RP7	1.19	W	A	F	C	E	G
RP8	1.16	W	A	F	C	E	G
S1	1.24	W	T	A	S	E	S
S2	1.30	W	T/A	A	S/C	E	S

^a^Amino acid residue numbers correspond to previously published sequences from *Arabidopsis thaliana* var. Columbia (Sathasivan et al. 1990). Listing of multiple amino acids at a particular number corresponds to a plant having different alleles of the ALSa or ALSb gene

^b^Amino acid abbreviations were according to UPAC 1-letter code for amino acids as follows: alanine (A), threonine (T), serine (S), phenylalanine (F), cysteine (C), glutamic acid (E), tryptophan (W), aspartic acid (D), glycine (G)

For mutation analysis, sequencing reads in each sample were mapped on gene contigs of *psb*A, ALSa, and ALSb with TopHat2 (Kim et al. 2013). Allelic differences were called as single nucleotide polymorphisms (SNPs) using the SAMtools software package (Li et al. 2009). The SNPs obtained were screened for polymorphisms resulting in amino acid changes associated with resistance to both ALS and PSII inhibiting herbicides.

## Recombinant expression, purification and in vitro inhibition studies of ALS

Effects of SNPs identified in sequencing analysis on ALS activity were studied using *A. thaliana.* The ALS gene from *A. thaliana* and derivatives thereof were de novo synthesized and subcloned into pET-24d(+) (Life Technologies. Grand Island, NY, USA) using NcoI and XhoI restriction sites. Protein constructs contained an 85 amino acid N-terminal deletion (chloroplast transit peptide) and the addition of an N-terminal Hexa-histidine tag to facilitate purification. Expression was performed as described by Chang and Duggleby (1997).

### Purification of recombinant ALS proteins

Cell pellets were re-suspended in Lysis buffer containing 10 mM phosphate buffer pH 7.5, 5 mM MgCl_2_, 10 mM imidazole, 150 mM NaCl, 1 mM EDTA, 10 % glycerol and one Complete EDTA-free proteolytic enzyme inhibition tablet (Roche Diagnostics Corporation. Indianapolis, IN). Lysis was performed by at least two passes through a fluidizer, followed by centrifuging at 80000×*g* for 30 min at 4 °C. The cleared lysate was loaded on Ni–NTA Superflow resin (Qiagen GmbH. Düsseldorf, Germany) pre-equilibrated with wash buffer (10 mM phosphate buffer pH 7.5, 5 mM MgCl_2_, 10 mM imidazole, 150 mM NaCl, 1 mM EDTA, 10 % glycerol), and washed with at least 20 column volumes of wash buffer. Elution was afforded with five column volumes of elution buffer (10 mM phosphate buffer pH 7.5, 5 mM MgCl_2_, 300 mM imidazole, 150 mM NaCl, 1 mM EDTA, 10 % glycerol) and collected as 1.5 ml fractions. Fractions containing ALS protein were pooled and stored at −20 °C.

### Assay of ALS activity and inhibition studies

ALS activity was assayed in a 140 µL reaction mixture containing DMSO (4 %vV), DTT (1 mM), PIPES (50 mM), MnCl_2_ (1 mM), FAD (0.02296 mM), cocarboxylase (15.4 µg), sodium pyruvate (40 mM) and enzyme (dissolved in 10 mM K_2_HPO_4_/KH_2_PO_4_, 5 mM MgCl_2_, 10 mM imidazole, 150 mM NaCl, 1 mM EDTA, 10 % glycerol) adjusted to pH 7.0. Inhibitors, purchased from Sigma as Pestanal standards (Sigma-Aldrich, Deisenhofen, Germany), were dissolved in DMSO and added to the reaction mixture. Inhibitors evaluated included imidazolinone, sulfonylurea, triazolopyrimidines, sulfonylamino-carbonyl- triazolinones, and pyrimidinyl (thio) benzoate herbicides. The reaction mixture was incubated at 30 °C for 60 min and stopped with 70 µl of H_2_SO_4_ (3 M). The reaction was then heated to 56 °C for 15 min to convert acetolactate into acetoin. The acetoin formed was quantified by incubation with 140 µL 1-naphtol mixture (1-naphtol (3.5 %, w/v), creatin (0.15 %, w/v), NaOH (19.25 %, w/v)) for 35 min at 56 °C, followed by an absorption measurement at 520 nm (eM = 22700 M^−1^ cm^−1^, determined using authentic acetoin). Inhibitor concentrations (M) required to reduce ALS activity by 50 % (IC_50_ values) were estimated using non-linear regression procedures. For each inhibitor, resistance factors were calculated by dividing IC_50_ values for mutant and sensitive ALS.

## Whole plant experiments

Progeny of all POAAN-R3 plants subjected to genetic sequencing (RP1–RP8) were surface seeded into separate 10.2 × 10.2 cm greenhouse pots (Dillen Products/Myers Industries, Inc. Middlefield, OH, USA.) filled with a peat moss growing medium (ProMix BX Mycorrhizae. Premier Tech Horticulture, Quakertown, PA, USA) on 5 January 2015. The susceptible annual bluegrass population (S) included in our sequencing analysis was also seeded in the same fashion.

After seeding, pots were placed inside a growth chamber (Conviron A1000. Controlled Environments Inc, Hendersonville, NC) configured to provide 14 h day at 19 °C and a 10 h night at 10 °C. Light intensity inside the chamber averaged 296 μmol m^−2^ s^−1^. Irrigation was applied to each pot daily using distilled water and a misting nozzle. Annual bluegrass emergence was first noted on 16 January 2015. Plants remained inside the growth chamber until 26 January 2015. On this date, seedlings of each plant (RP1–RP8, S) were transplanted into individual 164 cm^3^ plastic conical containers (SC10 Super Cell Cone-tainer. Stuewe and Sons. Tangent, OR 97389) filled with Sequatchie silt loam (fine-loamy, siliceous, semiactive, thermic humic Hapludult) measuring 6.2 in soil pH and 2.1 % in organic matter content. Transplants were replicated such that 32 plastic conical containers were created for each annual bluegrass plant analyzed in previous experiments (RP1–RP8, S). Immediately after transplanting, plastic conical containers were placed in a glasshouse located at the University of Tennessee (Knoxville, TN, USA; 35.56, 83.56). Peak light intensity in this glasshouse averaged 714 μmol m^−2^ s^−1^ with day/night temperatures averaging 27/17 °C. Irrigation was applied daily using an overhead misting system and plants were not clipped prior to initiating this study.

### Confirmation of target-site resistance

Plants were allowed to acclimate to this glasshouse environment for 10 days prior to being treated with foramsulfuron (Revolver. Bayer Environmental Sciences. Research Triangle Park, NC, USA) at 29 g ha^−1^, imazamox (Clearcast. BASF Corporation. Research Triangle Park, NC) at 140 g ha^−1^, or simazine (Princep 4L. Syngenta Professional Products. Greensboro, NC) at 1120 g ha^−1^. Per label recommendations, imazamox and simazine treatments included a non-ionic surfactant (Activator-90. Loveland Products Inc. Greeley, CO, USA) at 0.25 % v/v. All treatments were applied in an enclosed spray chamber (Generation III track sprayer. DeVries Manufacturing, Hollandale, MN, USA) using a water carrier at 215 L ha^−1^ through an 8004 EVS nozzle (TeeJet, Technologies, Spraying Systems Co., Glendale Heights, IL, USA). These herbicide treatments were selected to confirm results of genetic sequencing that RP1–RP8 were resistant to sulfonylurea, imidazolinone, and photosystem II inhibiting herbicides. A non-treated control treatment was included with each resistant (RP1–RP8) and susceptible (S) plant for comparison. Herbicides were applied on 5 February 2015 when plants were at a two-to-three leaf stage, prior to annual bluegrass tiller formation.

Annual bluegrass control was visually assessed on a 0 (i.e., no control) to 100 % (i.e., complete kill) scale relative to the non-treated 28 days after treatment. After all control data had been collected, aboveground biomass was harvested from each plastic conical container at the soil line using scissors, dried at 100 °C for 3 days, and weighed.

Treatments were arranged as a 9 × 4 factorial with nine plant selections (i.e., RP1–RP8, S) and four herbicide treatments (i.e., foramsulfuron, imazamox, simazine, non-treated) in a completely randomized design with four replications and repeated in time and space during February 2015. Plants were the same age during both experimental runs.

### Whole plant investigations of non-target site resistance to ALS inhibitors

Additional experiments were conducted during March–April 2015 to determine if non-target site based mechanisms conferred resistance to ALS inhibitors in our annual bluegrass population, POAAN-R3. Plants (RP6, RP7, and S) were cultured in plastic conical containers as previously described and allowed to acclimate to the glasshouse for 18 days prior to initiating two experiments.

In the first experiment, resistant (RP6, RP7) and susceptible (S) plants were treated with foramsulfuron at 29 g ha^−1^, trifloxysulfuron (Monument. Syngenta Professional Products. Greensboro, NC, USA) at 27.8 g ha^−1^, imazaquin (Image. BASF Corporation. Research Triangle Park, NC) at 27.5 g ha^−1^, or bispyribac-sodium (Velocity. Valent USA Corporation. Walnut Creek, CA, USA) at 70 g ha^−1^. All herbicides were applied alone or in combination with either malathion (Spectrum Group. St. Louis, MO, USA) at 1000 g ha^−1^ or piperonyl butoxide (Sigma Aldrich. St. Louis, MO, USA) at 2100 g ha^−1^. Malathion and piperonyl butoxide (PBO) inhibit cytochrome P450 monooxygenases involved in metabolism of ALS inhibiting herbicides and are commonly used at the application rates tested herein to evaluate non-target site based resistance mechanisms in weeds (Burnet et al. 1994; Christopher et al. 1992; Yu and Powles 2014b; Rao et al. 1995). All herbicides were mixed in water carrier and included non-ionic surfactant at 0.25 % v/v. Malathion and PBO were mixed with methanol and de-ionized water (50:50) and applied 60 min prior to herbicide application using a previously described enclosed spray chamber on 13 February 2015. Plants had a minimum of one tiller on the date of treatment application and averaged 5 cm in height.

Annual bluegrass control was visually assessed on a 0 (i.e., no control) to 100 % (i.e., complete kill) scale relative to the non-treated 21 days after treatment. After all control data had been collected, aboveground biomass was harvested from each plastic conical container at the soil line using scissors, dried at 100 °C, and weighed. Treatments were arranged as a 3 × 5 × 3 factorial with three plant selections (RP6, RP7, S), five herbicides (foramsulfuron, trifloxysulfuron, imazaquin, bispyribac-sodium, non-treated) and three P450 inhibitor treatments (malathion, PBO, non-treated) in a completely randomized design with four replications and repeated in separate glasshouses during February 2015.

A second experiment was conducted to determine if non-target mechanisms could confer resistance to ALS inhibiting herbicides in our annual bluegrass population, POAAN-R3. ALS-resistant (RP1–RP8) and susceptible (S) plants were cultured in plastic conical containers under previously described glasshouse conditions for 28 days before being treated with foramsulfuron (29 g ha^−1^) or sulfometuron-methyl (Oust XP; Dupont Professional Products. Wilmington, DE, USA) at 105 g ai ha^−1^. Sulfometuron-methyl is an ALS inhibiting herbicide not metabolized by cytochrome P450 monooxygenases; therefore, it can be used to discern if weed populations survive ALS inhibiting herbicide treatment via target- or non-target site based mechanisms (Christopher et al. 1992). Per label recommendations, sulfometuron-methyl included non-ionic surfactant at 0.25 % v/v. Treatments were mixed in water carrier applied in an enclosed spray chamber, as previously described, on 13 March 2015. Plants had a minimum of two tillers on the date of treatment application and averaged 14 cm in height.

Annual bluegrass control was visually assessed on a 0 (i.e., no control) to 100 % (i.e., complete kill) scale relative to the non-treated 28 days after treatment. After all control data had been collected, plant height data were collected using a ruler placed at the soil surface of each plastic conical container and recording the distance to the tip of the bud leaf. Treatments were arranged as a 9 × 3 factorial with nine plant selections (RP1–RP8, S) and three herbicides (foramsulfuron, sulfometuron-methyl, non-treated) in a completely randomized design with four replications and repeated in separate glasshouses during March 2015.

All data from these whole plant experiments were subjected to ANOVA in SAS (SAS version 9.1; SAS Institute. Cary, NC, USA). No significant experimental run interactions were detected; therefore, data from each experimental run were combined with means separated using Fisher’s protected LSD at *α* = 0.05.

## Results and discussion

### Genetic sequencing

An allotetraploid, individual annual bluegrass plants contain different homeologs of the ALS gene with both missense mutations as well as those that confer resistance to ALS-inhibiting herbicides (McElroy et al. 2013)^.^ In the current study, we were able to detect two distinct gene homeologs encoding for ALS proteins (ALSa (KT346395) and ALSb (KT346396)) with a coding sequence identity of 96.2 and 98.0 % (Supplementary Information, Figure S2). When comparing our sequences to the recently published transcript sequence assemblies for *Poa supina* and *Poa infirma* (Chen et al. 2015), it was found that ALSa originated from *Poa supina* and ALSb originated from *Poa infirma*. Heterozygosity was detected for both ALS genes within our POAAN-R3 population with individual plants having two different expressed alleles of ALSa and ALSb, respectively. Within the set of analyzed plants, four SNP positions were identified in the full length coding sequence of ALSa of which three were silent mutations and one resulted in a Trp-574-Leu substitution known to confer broad spectrum resistance to ALS inhibiting herbicides (Table 1). However, this mutation was only present in one (RP6) of the eight resistant plants analyzed.

In total 19 SNPs were identified in the full length coding sequence of ALSb from which 14 were silent and five resulted in distinct amino acid substitutions at different positions (Table 1). Interestingly, two distinct SNPs at nucleotide position 205 of ALSb occurred simultaneously in seven resistant plants (RP1–RP5, RP7, RP8); however, none of them were present in susceptible plants. The codon change from susceptible to resistant plants was GCC to TTC resulting in an Ala-205-Phe substitution. It could not be tracked if the two mutations from GC to TT occurred in one mutation event or if both mutations occurred sequentially in the population. If the two mutations occurred in a sequential manner, plants with the codon TCC (serine) or GTC (valine) should also exist within or in close proximity to the POAAN-R3 population from which plants in this study were selected. Another amino acid substitution was identified in ALSb of only RP6; a synonymous substitution at position 420 from glutamic acid-to-aspartic acid in a heterozygous form. Further analysis of the variability at this position in susceptible annual bluegrass samples is needed as this is a highly conserved amino acid change that has not been described before in any other resistant weed species.

McElroy et al. (2013) identified fragments of two distinct gene sequences in ALS-resistant annual bluegrass that included seven silent and two missense mutations, including a Trp-574-Leu change associated with resistance to ALS-inhibiting herbicides. All mutations described by McElroy et al. (2013) could be tracked either to nucleotide differences between ALSa and ALSb or identified as allelic differences in ALSa or ALSb genes in our POAAN-R3 population analyzed herein (Supplementary Information; Table S1). The researchers suggested that ancestral copies of the ALS gene may be unexpressed within the genome, similar to what has been reported with *Brassica napus* (Ouellet et al. 1992). Yu and Powles (2014a) explained that in polyploid species such as annual bluegrass, gene copies may all be expressed or silenced at different levels; however ALS resistance alleles are generally dominant over wild-type susceptible alleles in cases of heterozygosity. In the current study, ALSa and ALSb were expressed in a 1.16–1.35 ratio (ALSa/ALSb; Table 1). This indicates that both ALS isoforms were expressed in POAAN-R3 and contribute to overall ALS activity. In the current study, the plastidial encoded *psbA* gene coding for the target site of PSII inhibiting herbicides was either detected as the susceptible or resistant form as well (Table 1).

Our previous research had demonstrated that all eight POAAN-R3 plants sequenced in this study were resistant to the PSII and ALS inhibiting herbicides, simazine and trifloxysulfuron, respectively (Brosnan et al. 2015). Seven of the eight POAAN-R3 plants herein had a Ser-264-Gly substitution on the *psbA* gene, the basis for target site resistance to PSII inhibiting herbicides (such as simazine) in annual bluegrass (Kelly et al. 1999; Perry et al. 2012). One plant within this population (RP6) did not have this substitution (Table 1). The seven POAAN-R3 plants possessing the Ser-264-Gly substitution conferring resistance to simazine had no known amino acid substitutions associated with resistance to sulfonylurea herbicides (such as trifloxysulfuron), particularly the well documented the Trp-574-Leu mutation (Table 1) (McElroy et al. 2013). However, these plants had an Ala-205-Phe substitution on ALS. Amino acid changes at this position have been associated with resistance to imidazolinone herbicides in several weeds (Ashigh and Tardif 2007; McNaughton et al. 2005; Thompson and Tar’an 2014; White et al. 2003) (Table 1). However, this is the first report of an Ala-205-Phe substitution conferring resistance to ALS inhibiting herbicides. Characteristics of POAAN-R3 to different ALS herbicides were investigated with recombinant enzyme assays and whole plant studies herein.

### Recombinant analysis of ALS expression and activity

*In vitro* enzyme activity of wild type and mutant ALS protein was studied using recombinant *A. thaliana* DNA. Constructs were made to model wild-type ALS protein sensitive to ALS inhibiting herbicides as well as all mutations detected in RP1–RP8 annual bluegrass (Table 2). Resistance factors (IC_50_ values) were calculated after exposing these constructs to increasing concentrations of imidazolinone, sulfonylurea, triazolopyrimidines, sulfonylamino-carbonyl- triazolinones, and pyrimidinyl (thio) benzoate herbicides (Table 2). With the exception of florasulam, all variants exhibited resistance to all herbicides tested supporting the conclusions from genetic sequencing work that target-site mechanisms confer ALS resistance in POAAN-R3. Interestingly, the previously discussed Ala-205-Phe substitution conferred broad-spectrum resistance to all families of ALS inhibitors tested (Table 2). Amino acid substitutions at position 205 on ALS have been reported to confer mainly imidazolinone resistance in weeds (Ashigh and Tardif 2007; McNaughton et al. 2005; Thompson and Tar’an 2014; White et al. 2003); however, these data are the first report of an Ala to Phe substitution at this position conferring broad spectrum resistance to ALS inhibitors (e.g.imidazolinone, sulfonylurea, triazolopyrimidines, sulfonylamino-carbonyl- triazolinones, and pyrimidinyl (thio) benzoate herbicides). In wild radish (*Raphanus raphanistrum*) an Ala-122-Tyr substitution endows broad spectrum resistance to ALS inhibitors (Han et al. 2012) whereas Ala-122-Thr mutation usually only confers resistance to the imidazolinone herbicides (Tranel and Wright 2002). These results indicate ALS mutations conferring cross resistance are determined not only by the site of the mutation but also by the specific amino acid change as well. Interestingly, the Ala-205-Phe substitution detected herein provided a higher level of resistance to sulfonylurea and sulfonylamino-carbonyl- triazolinone herbicides than a Trp-574-Leu substitution known to confer broad target site resistance in annual bluegrass (Table 2).

**Table 2:** Effects of acetolactate synthase (ALS) inhibitors on in vitro enzyme activity of recombinant *Arabidopsis thaliana* ALS wild-type enzyme (WT—sensitive) and variants (resistant), containing the amino acid substitutions identified in ALS resistant annual bluegrass RP1–RP8

Relative enzyme rate in absence of inhibitor^c^	WT sensitivity [IC_50_] (M)^a^	Resistance factor		
		A205V^b^	A205F	W574L
	2.8	0.63	0.7	1.44
Chemical family				
Imidazolinones				
Imazamox	2.93 × 10^−6^	34	34	34
Imazapyr	6.06 × 10^−6^	16	16	16
Imazaquin	1.55 × 10^−6^	36	19	65
Sulfonylureas				
Foramsulfuron	7.50 × 10^−9^	12	5160	2013
Trifloxysulfuron	5.90 × 10^−9^	9	2983	6447
Bensulfuron	4.23 × 10^−9^	54	23,657	642
Prosulfuron	2.88 × 10^−9^	88	34,756	3524
Tritosulfuron	1.87 × 10^−8^	37	5356	167
Nicosulfuron	7.14 × 10^−8^	30	1401	35
Primisulfuron	3.14 × 10^−9^	429	31,803	5728
Mesosulfuron	1.33 × 10^−9^	16	293	104
Triazolopyrimidines				
Pyroxulam	4.12 × 10^−9^	162	24,268	24,268
Metosulam	4.41 × 10^−10^	34	1656	107
Florasulam	3.43 × 10^−9^	89	1	370
Sulfonylamino-carbonyl- triazolinones				
Flucarbazone	2.45 × 10^−8^	2	441	7
Propoxycarbazone	9.28 × 10^−9^	20	1185	309
Thiencarbazone	2.48 × 10^−8^	8	62	16
Pyrimidinyl (thio) benzoates				
Bispyribac	1.58 × 10^−9^	11	7	7944
Pyrithiobac	7.42 × 10^−10^	433	306	134,838
Pyriftalid	2.00 × 10^−5^	5	5	5

Resistance factors were calculated using concentrations of inhibitors required to reduce activity of the WT by 50 % (IC_50_ M). Resistance factors can only be compared within a chemical family. Maximum inhibitor concentration used to determine IC_50_ was 1.00 × 10^−04^ M

^a^Concentration [M] required for 50 % inhibition after treatment with corresponding inhibitor

^b^Amino acid residue numbers correspond to previously published sequences from *Arabidopsis thaliana* var. Columbia (Sathasivan et al. 1990)

^c^Represent the enzyme activity as an arbitrary unit to show the relative activity of the mutants compared to the wildtype

### Whole plant confirmation of target-site resistance

As expected, significant differences in annual bluegrass control were observed 28 days after treating resistant (RP1–RP8) and susceptible (S) annual bluegrass with foramsulfuron and imazamox (Table 3). With the exception of RP6, neither foramsulfuron nor imazamox resulted in >11 % annual bluegrass control. Relative to non-treated plants, resistant selections treated with these herbicides yielded 41 to 94 % biomass compared to 0 to 3 % for those susceptible to ALS inhibitors (Supplementary Information; Table S2). These whole plant responses support conclusions from sequencing and in vitro activity assays that RP1–RP5, RP7, and RP8 plants contain an alanine-to-phenylalanine substitution on ALS that confers resistance to ALS inhibiting herbicides. RP6 plants do not contain this substitution (Table 1). Rather, RP6 plants were heterozygous for Trp-574-Leu substitution on ALSa (Table 1). While RP6 plants were also resistant to foramsulfuron and imazamox (16–24 % control), they were more sensitive than those with Ala-205-Phe mutation (Table 3). This whole plant response supports results of in vitro assays that determined Ala-205-Phe substitution conferred 5160-fold resistance to foramsulfuron compared to 2013-fold resistance for the Trp-574-Leu mutation (Table 2).

**Table 3:** Annual bluegrass (*Poa annua* L.) control 28 days after treatment with foramsulfuron (29 g ha^−1^), imazamox (140 g ha^−1^), and simazine (1120 g ha^−1^) to herbicide resistant (RP1–RP8) and susceptible (S) plants at a 2 to 3 leaf stage

Herbicide^a^	Rate (g ha^−1^)	Annual bluegrass control								
		RP1 (%)	RP2 (%)	RP3 (%)	RP4 (%)	RP5 (%)	RP6 (%)	RP7 (%)	RP8 (%)	S (%)
Foramsulfuron	29	8	11	11	5	10	24	10	5	93
Imazamox	140	2	0	0	0	3	16	4	0	93
Simazine	1120	0	0	0	0	0	100	0	0	98
LSD_0.05_		5	5	5	4	7	23	8	NS	7

Means below were combined from two experimental runs conducted under glasshouse conditions in Knoxville, TN during February 2015

^a^Per label recommendations, imazamox and simazine were mixed with non-ionic surfactant (Activator-90. Loveland Products Inc. Greeley, CO, USA) at 0.25 % v/v

All resistant plants analyzed except RP6 contained *psbA* Ser-264-Gly mutations (Table 1). Simazine failed to control these plants in whole plant experiments, while plants without this amino acid substitution (RP6, S) were controlled 98 to 100 % (Table 3). Coupled with our sequencing analysis, this finding indicates that resistance to simazine in POAAN-R3 is target-site based. Resistant plants treated with simazine also yielded dry biomass values ranging from 82 to 140 % of the non-treated control (Supplementary Table S2). Brosnan et al. (2015) reported a similar effect following preemergence applications of simazine to POAAN-R3 and suggested the response may be due to hormesis. Herbicide resistant weeds can be subject to hormesis following continued use of the same herbicide at labeled rates without rotation (Calabrese and Baldwin 2002; Belz et al. 2011). Hormesis is a plausible explanation for the increased biomass observed herein considering that POAAN-R3 was originally harvested from a golf course that applied simazine for annual bluegrass control for 20 consecutive years (Brosnan et al. 2015).

### Whole plant investigations of non-target site resistance

To evaluate the potential for non-target site resistance mechanisms within POAAN-R3, two resistant plants with varying amino acid substitutions on ALS (RP6, RP7; Table 1) were treated with sulfonylurea, imidazolinone, and pyrimidinyl (thio) benzoate herbicides alone and following treatment with cytochrome P450 inhibitors malathion and PBO. Overall levels of annual bluegrass control 21 days after treatment were quite low for all treatment combinations, further supporting that ALS resistance in POAAN-R3 is target site based. For example, foramsulfuron and trifloxysulfuron resulted in 24 to 58 % control compared to 91 to 96 % for the susceptible population (Table 4). With the exception of bispyribac-sodium application to RP7, neither malathion nor PBO increased control of resistant annual bluegrass following herbicide application. Given that malathion and PBO are known inhibitors of cytochrome P450 enzymes involved in ALS herbicide metabolism, these data indicate that non-target site based resistance mechanisms are likely not present in POAAN-R3.

**Table 4:** Annual bluegrass (*Poa annua* L.) control 21 days after treatment with foramsulfuron (29 g ha^−1^), trifloxysulfuron (27.8 g ha^−1^), imazaquin (27.5 g ha^−1^), or bispyribac-sodium (70 g ha^−1^) alone in combination with malathion (1000 g ha^−1^) or piperonyl butoxide (2100 g ha^−1^)

Herbicide^a^	P450 inhibitor^b^	Rate (g ha^−1^)	Annual bluegrass control		
			RP6 (%)	RP7 (%)	S (%)
Foramsulfuron	–	29	58	28	91
	Malathion	1000	24	22	96
	PBO	2100	31	24	95
			*	NS	NS
Trifloxysulfuron	–	27.8	38	22	94
	Malathion	1000	49	28	92
	PBO	2100	55	17	94
			NS	NS	NS
Imazaquin	–	27.5	36	11	53
	Malathion	1000	32	8	64
	PBO	2100	24	9	71
			NS	NS	*
Bispyribac-sodium	–	74	28	7	90
	Malathion	1000	32	47	92
	PBO	2100	20	14	86
*P* < *F*			NS	*	NS
		LSD	22	14	11

Treatments were applied to ALS resistant (RP6, RP7) and susceptible (S) annual bluegrass with a minimum of one tiller and average height of 5 cm. Means below were combined from two experimental runs conducted under glasshouse conditions in Knoxville, TN during February 2015

^a^All herbicides were mixed with non-ionic surfactant (Activator-90. Loveland Products Inc. Greeley, CO, USA) at 0.25 % v/v

^b^Malathion and piperonyl butoxide (PBO) were applied 60 min prior to herbicide treatment in a solution of methanol and de-ionized water (50:50)

* Significantly different from one another at α = 0.05

Sulfometuron, a herbicide not subject to cytochrome P450 metabolism, failed to effectively control POAAN-R3 in whole plant assays (Table 5). With the exception of RP6, foramsulfuron and sulfometuron controlled resistant plants 2 to 29 % compared to 93 to 99 % for a susceptible population (Table 5). This further indicates that the Ala-205-Phe substitution identified in RP1–RP5, RP7, and RP8 (Table 1) is a new amino acid mutation on ALS that confers broad resistance to ALS inhibiting herbicides. *In vitro* assays with recombinant ALS protein indicated that this Ala-205-Phe substitution confers a higher level of resistance to foramsulfuron than a Trp-574-Leu substitution (Table 3). Whole plant data in these experiments support that conclusion as foramsulfuron controlled RP6 (a plant with a Trp-574-Leu substitution) 46 % compared to ≤11 % control for RP1–RP5, RP7, and RP8 plants that contained Ala-205-Phe substitutions.

**Table 5:** Annual bluegrass (*Poa annua* L.) control 28 days after treatment with foramsulfuron (29 g ha^−1^), and sulfometuron (105 g ha^−1^) to herbicide resistant (RP1–RP8) and susceptible (S) plants with a minimum of two tillers

Herbicide^a^	Rate (g ha^−1^)	Annual bluegrass control								
		RP1 (%)	RP2 (%)	RP3 (%)	RP4 (%)	RP5 (%)	RP6 (%)	RP7 (%)	RP8 (%)	S (%)
Foramsulfuron	29	8	2	5	11	8	46	5	3	93
Sulfometuron	105	24	29	21	21	26	76	28	21	99
LSD_0.05_		15	4	8	NS	7	NS	7	10	NS

Means below were combined from two experimental runs conducted under glasshouse conditions in Knoxville, TN during March 2015

^a^Per label instructions, sulfometuron was mixed with non-ionic surfactant (Activator-90. Loveland Products Inc. Greeley, CO, USA) at 0.25 % v/v

## Conclusion

Two gene isoforms encoding for ALS were detected within individual annual bluegrass plants in the POAAN-R3 population, both of which were equally expressed and contributed to ALS activity. Amino acid changes in these isoforms conferring resistance to ALS and PSII inhibiting herbicides varied among selections within the POAAN-R3 population. Seven resistant plants analyzed had Ser-264-Gly mutations that conferred resistance to simazine in whole plant assays, similar to reports by Brosnan et al. (2015). These same seven plants also had Ala-205-Phe substitutions on ALS that conferred broad spectrum in vitro resistance to imidazolinone, sulfonylurea, triazolopyrimidines, sulfonylamino-carbonyl- triazolinones, and pyrimidinyl (thio) benzoate herbicides when expressed in *Arabidopsis* and to sulfonylurea, imidazolinone, and pyrimidinyl (thio) benzoate herbicides at the whole plant level. One plant within POAAN-R3 had neither a Ser-264-Gly mutation on *psbA* nor an Ala-205-Phe mutation on ALS. However, this plant was heterozygous for a Trp-574-Leu mutation on a different ALS isoform than we observed the Ala-205-Phe substitution. Consequently, this plant (RP6) was susceptible to simazine treatment and more sensitive to sulfonylurea herbicides than plants containing the Ala-205-Phe substitution.

Previous research had surmised that non-target mechanisms in POAAN-R3 may confer resistance to ALS inhibiting herbicides (Brosnan et al. 2015). Data presented herein contradict that assertion. Considering that multiple ALS isoforms were detected in POAAN-R3 plants, it is possible that measuring ALS activity using acetolactate protein extracted from whole plants, similar to Brosnan et al. (2015), may not be appropriate for polyploid species such as annual bluegrass. In the absence of herbicide, overall ALS activity with Ala-205-Phe mutation was approximately 75 % lower than susceptible plants and 50 % lower than resistant plants with Trp-574-Leu substitutions (Table 2). Future research should evaluate the appropriateness of extractable ALS activity assays on polyploid weed species resistant to ALS inhibitors.

Overall, our findings illustrate the presence and expression of two ALS isoforms in POAAN-R3, a population of annual bluegrass resistant to ALS and PSII inhibiting herbicides. One of these ALS isoforms contained Ala-205-Phe mutation. In vitro enzyme assays with recombinant *A. thaliana* ALS protein and whole plant experiments confirmed that this mutation conferred broad spectrum resistance to multiple herbicide families targeting ALS. This is the first report of an alanine-to-phenylalanine substitution conferring resistance to imidazolinone, sulfonylurea, triazolopyrimidines, sulfonylamino-carbonyl- triazolinones, and pyrimidinyl (thio) benzoate herbicides. Interestingly, this substitution is based on two nucleic acid substitutions and it could not be determined if these were the result of two consecutive mutation events or two mutations that occurred concurrently. A more detailed investigation of the population should be envisaged to check if an alanine-to-valine substitution in ALS is also present in the population, which would be an indication for two consecutive mutation events during the evolution process.

### *Author contribution statement*

James T. Brosnan, Jose J. Vargas, and Gregory Breeden collected plant material from the field and cultured plants from laboratory and glasshouse experiments. Additionally, these researchers designed and conducted all glasshouse experiments presented herein. Logan Grier, Raphael A. Aponte, Stefan Tresch, and Martin Laforest designed and conducted all sequencing and laboratory assay experiments. James T. Brosnan, Raphael A. Aponte, Stefan Tresch, and Martin Laforest shared responsibility in preparing the manuscript based on their contributions to the research.

## Electronic supplementary material

Supplementary material 1 (DOCX 58 kb)
