# Supplementary material for: A new amino acid substitution (Ala-205-Phe) in acetolactate synthase (ALS) confers broad spectrum resistance to ALS-inhibiting herbicides
Source: Planta. 2015 Sep 9;243:149–59. doi: 10.1007/s00425-015-2399-9 (PMC4698308; doi:10.1007/s00425-015-2399-9)
Supplement: Supplementary file 1 — Supplementary material 1 (DOCX 58 kb) [file 425_2015_2399_MOESM1_ESM.docx]

**Title**: A New Amino Acid Substitution (Ala-205-Phe) in Acetolactate Synthase Confers Broad Spectrum Resistance to Acetolactate Synthase Inhibiting Herbicides

**Authors:** James T. Brosnan^a*^, Jose J. Vargas^a^, Gregory K. Breeden^a^, Logan Grier^b^, Raphael A. Aponte^c^, Stefan Tresch^c^, and Martin Laforest^d^

**Supplementary Information**

Table S1. Complete list of single nucleotide polymorphisms identified in ALSa, ALSb and psbA (D1) genes of annual bluegrass resistant (RP1-P8) and susceptible (S1-S2) acetolactate synthase (ALS) and photosystem II inhibiting herbicides.

| Gene | NA position in *Poa annua* gene | NA position in reference gene (*A. thaliana*) | Reference nucleic acid | Alternative nucleic acid | mutation | AA position *Poa annua* protein | AA position in *A. thaliana* protein | Zygosity of SNPs^a^ | | | | | | | | | |
| --- | --- | --- | --- | --- | --- | --- | --- | --- | --- | --- | --- | --- | --- | --- | --- | --- | --- |
|  |  |  |  |  |  |  |  | S1 | S2 | RP1 | RP2 | RP3 | RP4 | RP5 | RP6 | RP7 | RP8 |
| Pa_ALS_a | 179 | 258 | C | G | silent |  |  | 0/0 | 0/1 | 0/0 | 0/0 | 0/0 | 0/0 | 0/0 | 0/0 | 0/0 | 0/0 |
| Pa_ALS_a | 1142 | 1221 | A | C | silent |  |  | 1/1 | 0/0 | 0/0 | 0/0 | 0/0 | 0/0 | 0/0 | 0/0 | 0/0 | 0/0 |
| Pa_ALS_a | 1580 | 1656 | T | C | silent |  |  | 1/1 | 0/1 | 0/0 | 0/0 | 0/0 | 0/0 | 0/0 | 0/1 | 0/0 | 0/0 |
| Pa_ALS_a | 1645^b^ | 1721 | G | T | Trp --> Leu | 542 | 574 | 0/0 | 0/0 | 0/0 | 0/0 | 0/0 | 0/0 | 0/0 | 0/1 | 0/0 | 0/0 |
| Pa_ALS_b | 185 | 231 | C | G | silent |  |  | 0/0 | 0/1 | 1/1 | 1/1 | 1/1 | 1/1 | 1/1 | 1/1 | 1/1 | 1/1 |
| Pa_ALS_b | 219 | 280 | A | G | Thr/Ala | 66 | 94 | 0/0 | 0/1 | 1/1 | 1/1 | 1/1 | 1/1 | 1/1 | 1/1 | 1/1 | 1/1 |
| Pa_ALS_b | 296 | 357 | C | G | silent |  |  | 0/0 | 0/0 | 1/1 | 1/1 | 1/1 | 1/1 | 1/1 | 1/1 | 1/1 | 1/1 |
| Pa_ALS_b | 552 | 613 | G | T | Ala --> Phe | 177 | 205 | 0/0 | 0/0 | 1/1 | 1/1 | 1/1 | 1/1 | 1/1 | 0/0 | 1/1 | 1/1 |
| Pa_ALS_b | 553 | 614 | C | T |  |  |  | 0/0 | 0/0 | 1/1 | 1/1 | 1/1 | 1/1 | 1/1 | 0/0 | 1/1 | 1/1 |
| Pa_ALS_b | 611 | 672 | G | C | silent |  |  | 0/0 | 0/1 | 1/1 | 1/1 | 1/1 | 1/1 | 1/1 | 1/1 | 1/1 | 1/1 |
| Pa_ALS_b | 617 | 678 | C | T | silent |  |  | 0/0 | 0/1 | 1/1 | 1/1 | 1/1 | 1/1 | 1/1 | 1/1 | 1/1 | 1/1 |
| Pa_ALS_b | 806 | 867 | T | C | silent |  |  | 0/0 | 0/0 | 1/1 | 1/1 | 1/1 | 1/1 | 1/1 | 1/1 | 1/1 | 1/1 |
| Pa_ALS_b | 867 | 928 | A | T | Ser/Cys | 282 | 310 | 0/0 | 0/1 | 1/1 | 1/1 | 1/1 | 1/1 | 1/1 | 1/1 | 1/1 | 1/1 |
| Pa_ALS_b | 995 | 1056 | T | C | silent |  |  | 0/0 | 0/1 | 1/1 | 1/1 | 1/1 | 1/1 | 1/1 | 1/1 | 1/1 | 1/1 |
| Pa_ALS_b | 1010 | 1071 | G | C | silent |  |  | 0/0 | 0/0 | 1/1 | 1/1 | 1/1 | 1/1 | 1/1 | 1/1 | 1/1 | 1/1 |
| Pa_ALS_b | 1199 | 1260 | G | T | Glu --> Asp | 392 | 420 | 0/0 | 0/0 | 0/0 | 0/0 | 0/0 | 0/0 | 0/0 | 0/1 | 0/0 | 0/0 |
| Pa_ALS_b | 1214 | 1275 | C | T | silent |  |  | 0/0 | 0/0 | 1/1 | 1/1 | 1/1 | 1/1 | 1/1 | 1/1 | 1/1 | 1/1 |
| Pa_ALS_b | 1301 | 1359 | A | G | silent |  |  | 0/0 | 0/1 | 1/1 | 1/1 | 1/1 | 1/1 | 1/1 | 1/1 | 1/1 | 1/1 |
| Pa_ALS_b | 1334 | 1391 | A | G | silent |  |  | 1/1 | 0/1 | 0/0 | 0/0 | 0/0 | 0/0 | 0/0 | 0/0 | 0/0 | 0/0 |
| Pa_ALS_b | 1352 | 1410 | A | G | silent |  |  | 1/1 | 0/1 | 0/0 | 0/0 | 0/0 | 0/0 | 0/0 | 0/0 | 0/0 | 0/0 |
| Pa_ALS_b | 1706* | 1764 | T | G | silent |  |  | 0/0 | 0/0 | 1/1 | 1/1 | 1/1 | 1/1 | 1/1 | 1/1 | 1/1 | 1/1 |
| Pa_ALS_b | 1796* | 1854 | G | A | silent |  |  | 0/0 | 0/0 | 1/1 | 1/1 | 1/1 | 1/1 | 1/1 | 1/1 | 1/1 | 1/1 |
| Pa_ALS_b | 1823 | 1881 | A | G | silent |  |  | 0/0 | 0/0 | 1/1 | 1/1 | 1/1 | 1/1 | 1/1 | 1/1 | 1/1 | 1/1 |
| Pa_psbA | 311 |  | G | T | silent |  |  | 0/0 | 0/1 | 0/0 | 0/0 | 0/0 | 0/0 | 0/0 | 0/0 | 0/0 | 0/0 |
| Pa_psbA | 453 |  | C | T | silent |  |  | 1/1 | 0/0 | 0/0 | 0/0 | 0/0 | 0/0 | 0/0 | 0/0 | 0/0 | 0/0 |
| Pa_psbA | 903 |  | A | G | Ser --> Gly | 264 | 264 | 0/0 | 0/0 | 1/1 | 1/1 | 1/1 | 1/1 | 1/1 | 0/0 | 1/1 | 1/1 |

^a^ Zygosity compared to reference nucleic acid: 0/0 homozygous for reference nucleic acid; 0/1 heterozygous, reference and alternative nucleic acid present; 1/1 homozygous for alternative nucleic acid

^b^ SNP also described in McElroy et. al. (2013). All other six SNPs identified in McElroy et al. (2013) based on differences between ALSa and ALSb gene sequences were detected herein.

Table S2. Annual bluegrass (*Poa annua* L.) biomass 28 days after treatment with foramsulfuron (29 g ha^-1^), imazamox (140 g ha^-1^), and simazine (1120 g ha^-1^) to herbicide resistant (RP1-RP8) and susceptible (S) plants at a 2 to 3 leaf stage. All aboveground tissue in each cone-tainer was harvested at the soil line, placed in a forced air drying oven at 100°C for 48 h and weighed. Means below were combined from two experimental runs conducted under glasshouse conditions in Knoxville, TN during February 2015

|  |  | Annual bluegrass biomass | | | | | | | | |
| --- | --- | --- | --- | --- | --- | --- | --- | --- | --- | --- |
| Herbicide^a^ | Rate | RP1 | RP2 | RP3 | RP4 | RP5 | RP6 | RP7 | RP8 | S |
|  | g ha^-1^ | ^_______________________________________^% of nontreated control^_______________________________________________^ | | | | | | | | |
| foramsulfuron | 29 | 60 | 45 | 48 | 41 | 62 | 49 | 52 | 51 | 0 |
| imazamox | 140 | 84 | 66 | 94 | 55 | 49 | 70 | 87 | 86 | 3 |
| simazine | 1120 | 124 | 105 | 140 | 95 | 82 | 0 | 99 | 101 | 5 |
| LSD_0.05_ | | 26 | 19 | 50 | 19 | NS | 51 | 35 | 39 | NS |

^a^ Per label recommendations, imazamox and simazine were mixed with non-ionic surfactant (Activator-90. Loveland Products Inc. Greely, CO) at 0.25% v/v.

Table S3. Annual bluegrass (*Poa annua* L.) plant height 28 days after treatment with foramsulfuron (29 g ha^-1^), and sulfometuron (105 g ha^-1^) to herbicide resistant (RP1-RP8) and susceptible (S) plants with a minimum of two tillers. Means below were combined from two experimental runs conducted under glasshouse conditions in Knoxville, TN during March 2015.

|  |  | Annual bluegrass height^a^ | | | | | | | | |
| --- | --- | --- | --- | --- | --- | --- | --- | --- | --- | --- |
| Herbicide^b^ | Rate | RP1 | RP2 | RP3 | RP4 | RP5 | RP6 | RP7 | RP8 | S |
|  | g ha^-1^ | ^___________________________________________________________^mm^____________________________________________________________^ | | | | | | | | |
| foramsulfuron | 29 | 77 | 74 | 100 | 85 | 75 | 68 | 75 | 94 | 18 |
| sulfometuron | 105 | 76 | 65 | 78 | 71 | 68 | 56 | 69 | 67 | 9 |
| Non-treated |  | 118 | 129 | 173 | 140 | 114 | 106 | 125 | 132 | 111 |
| LSD_0.05_ | | 16 | 19 | 26 | 11 | 12 | 29 | 9 | 14 | 20 |

^a^ Plant height data were collected using a ruler measuring the distance from the soil surface in each cone-tainer to the tip if the bud leaf.

^b^ Per label instructions, sulfometuron was mixed with non-ionic surfactant (Activator-90. Loveland Products Inc. Greely, CO) at 0.25% v/v.

Figure S1. Amino acid alignment of *Arabidopsis thaliana* and *Poa annua* ALS proteins (ALSa and ALSb)

* 20 * 40 * 60 *
At_ALS : MAAATTTTTTSSSISFSTKPSPSSSKSPLPISRFSLPFSLNPNKSSSSSRRRGIKSSSPSSISAVLNTTT : 70
Pa_ALSa : ---MA.A...AVA..GA.SAL.KP.----------..RH.PAS.R-------------------A.AA.R : 38
Pa_ALSa_R6 : ---MA.A...AVA..GA.SAL.KP.----------..RH.PAS.R-------------------A.AA.R : 38
Pa_ALSb : -M.TA.A...AVA..GA.SAL.KP.----------..RH.PAW.RA-----------------..AAA.R : 42
Pa_ALSb_Rx : -M.TA.A...AVA..GA.SAL.KP.----------..RH.PAW.RA-----------------..AAA.R : 42
Pa_ALSb_R6 : -M.TA.A...AVA..GA.SAL.KP.----------..RH.PAW.RA-----------------..AAA.R : 42
 ××× × × ×××
 80 * 100 * 120 * 140
At_ALS : NVTTTPSPTKPTKPETFISRFAPDQPRKGADILVEALERQGVETVFAYPGGASMEIHQALTRSSSIRNVL : 140
Pa_ALSa : IRC..V.-PS.AP.A.A.RP.G.T...............C..SD...................PA.T.H. : 107
Pa_ALSa_R6 : IRC..V.-PS.AP.A.A.RP.G.T...............C..SD...................PA.T.H. : 107
Pa_ALSb : IRC..V.SPS.AP.A.A.RP.G.T...............C..SD...................PA.T.H. : 112
Pa_ALSb_Rx : IRC..V.SPS.AP.A.A.RP.G.A...............C..SD...................PA.T.H. : 112
Pa_ALSb_R6 : IRC..V.SPS.AP.A.A.RP.G.T...............C..SD...................PA.T.H. : 112
 × æ
 * 160 * 180 * 200 *
At_ALS : PRHEQGGVFAAEGYARSSGKPGICIATSGPGATNLVSGLADALLDSVPLVAITGQVPRRMIGTDAFQETP : 210
Pa_ALSa : F.....EA...S....A...V................A................................ : 177
Pa_ALSa_R6 : F.....EA...S....A...V................A................................ : 177
Pa_ALSb : F.....EA...S....A...V................A................................ : 182
Pa_ALSb_Rx : F.....EA...S....A...V................A..........................F..... : 182
Pa_ALSb_R6 : F.....EA...S....A...V................A................................ : 182
 µ
 220 * 240 * 260 * 280
At_ALS : IVEVTRSITKHNYLVMDVEDIPRIIEEAFFLATSGRPGPVLVDVPKDIQQQLAIPNWEQAMRLPGYMSRM : 280
Pa_ALSa : .......................................................V.DAP.S.....A.. : 247
Pa_ALSa_R6 : .......................................................V.DAP.S.....A.. : 247
Pa_ALSb : .......................................................V.DAP.S.....A.. : 252
Pa_ALSb_Rx : .......................................................V.DAP.S.....A.. : 252
Pa_ALSb_R6 : .......................................................V.DAP.S.....A.. : 252

 * 300 * 320 * 340 *
At_ALS : PKPPEDSHLEQIVRLISESKKPVLYVGGGCLNSSDELGRFVELTGIPVASTLMGLGSYPCDDELSLHMLG : 350
Pa_ALSa : ....ATEL........G.A...........SA.GE..R..........T.......N..S..P...R... : 317
Pa_ALSa_R6 : ....ATEL........G.A...........SA.GE..R..........T.......N..S..P...R... : 317
Pa_ALSb : ....ATEL........G............SSA.GE..R..........T.......N..S..P...R... : 322
Pa_ALSb_Rx : ....ATEL........G.............SA.GE..R..........T.......N..S..P...R... : 322
Pa_ALSb_R6 : ....ATEL........G............SSA.GE..R..........T.......N..S..P...R... : 322
 × æ
 360 * 380 * 400 * 420
At_ALS : MHGTVYANYAVEHSDLLLAFGVRFDDRVTGKLEAFASRAKIVHIDIDSAEIGKNKTPHVSVCGDVKLALQ : 420
Pa_ALSa : ...........DKA........................S........P.......Q......A....... : 387
Pa_ALSa_R6 : ...........DKA........................S........P.......Q......A....... : 387
Pa_ALSb : ...........DKA........................S........P.......Q......A....... : 392
Pa_ALSb_Rx : ...........DKA........................S........P.......Q......A....... : 392
Pa_ALSb_R6 : ...........DKA........................S........P.......Q......A......D : 392
 µ
 * 440 * 460 * 480 *
At_ALS : GMNKVLEN-RAEELKLDFGVWRNELNVQKQKFPLSFKTFGEAIPPQYAIKVLDELTDGKAIISTGVGQHQ : 489
Pa_ALSa : ...S..L.GSKTHKS...SS.HE...Q..RE...G..............Q......K.E...A....... : 457
Pa_ALSa_R6 : ...S..L.GSKTHKS...SS.HE...Q..RE...G..............Q......K.E...A....... : 457
Pa_ALSb : ...S..L.GSKTHKS...SS.HE...Q..RE...G..............Q......K.E...A....... : 462
Pa_ALSb_Rx : ...S..L.GSKTHKS...SS.HE...Q..RE...G..............Q......K.E...A....... : 462
Pa_ALSb_R6 : ...S..L.GSKTHKS...SS.HE...Q..RE...G..............Q......K.E...A....... : 462

 500 * 520 * 540 * 560
At_ALS : MWAAQFYNYKKPRQWLSSGGLGAMGFGLPAAIGASVANPDAIVVDIDGDGSFIMNVQELATIRVENLPVK : 559
Pa_ALSa : .......T..........A............A..A....GVT..................L......... : 527
Pa_ALSa_R6 : .......T..........A............A..A....GVT..................L......... : 527
Pa_ALSb : .......T..........A............A..A....GVT..................L......... : 532
Pa_ALSb_Rx : .......T..........A............A..A....GVT..................L......... : 532
Pa_ALSb_R6 : .......T..........A............A..A....GVT..................L......... : 532

 * 580 * 600 * 620 *
At_ALS : VLLLNNQHLGMVMQWEDRFYKANRAHTFLGDPAQEDEIFPNMLLFAAACGIPAARVTKKADLREAIQTML : 629
Pa_ALSa : ................................EN.S.....F.TI.KGFN...V.....SE..A..KK.. : 597
Pa_ALSa_R6 : ..............L.................EN.S.....F.TI.KGFN...V.....SE..A..KK.. : 597
Pa_ALSb : ................................EN.S.....F.TI.KGFN...V.....SE..A..K... : 602
Pa_ALSb_Rx : ................................EN.S.....F.TI.KGFN...V.....SE..A..K... : 602
Pa_ALSb_R6 : ................................EN.S.....F.TI.KGFN...V.....SE..A..K... : 602
 µ ×
 640 * 660 *
At_ALS : DTPGPYLLDVICPHQEHVLPMIPSGGTFNDVITEGDGRIKY : 670
Pa_ALSa : E..........V..............A.K...MD.....A. : 638
Pa_ALSa_R6 : E..........V..............A.K...MD.....A. : 638
Pa_ALSb : E..........V..............A.K...M......A. : 643
Pa_ALSb_Rx : E..........V..............A.K...M......A. : 643
Pa_ALSb_R6 : E..........V..............A.K...M......A. : 643
 ×
Legend: Rx = ALSb variants expressed in herbicide resistant plants RP1-5, RP7, RP8; × = variation ALSa / ALSb expression ratio; µ = mutation of R plant versus S plant; æ = differences also detected in susceptible plants

Figure S2. Nucleic acid alignment of *Poa annua* (Pa) ALSa and ALSb genes, compared to nucleic acid sequences of ALS genes from *Poa supina* (Ps) and *Poa infirma* (Pi)

* 20 * 40 * 60 *
Pa_ALSa : ---------------------------------------------------------------------- : -
Ps_ALS : ---------------GGCCAAGATTGCGCTTGCGCTACAGCAGATCTCACACGGTCACACACACCCGGGA : 55
Pa_ALSb : ---------------------------------------------------------------------- : -
Pi_ALS : CGCTCGGAAGATTCAGCTGAAAAAAAAAGGTGCGGC-CAAGATTGCACAGCAGATCTCACATCACACGGA : 69

 80 * 100 * 120 * 140
Pa_ALSa : -----------------------------------------CTCC------------CGCCGCCGCCGCC : 17
Ps_ALS : TAAAAATCAAGATTCTCTCCTCTTTCTCCCTCGCCCAAACC....------------............. : 113
Pa_ALSb : --------------------------------------------------CCGCAAT...T......... : 20
Pi_ALS : TAAAAATCAAGATTCTCCTCTCTCTCTCCCTCGCCCAAACC....CGCCGCCGCAAT...T......... : 139

 * 160 * 180 * 200 *
Pa_ALSa : GCCATGGCCACAGCCAC------GTCCACAGCCGTCGCCATCTCGGGCGCCACCTCCGCCCTACCCAAAC : 81
Ps_ALS : .................------............................................... : 177
Pa_ALSb : .................AGCCAC.....................................T......... : 90
Pi_ALS : .................AGCCAC.....................................T......... : 209

 220 * 240 * 260 * 280
Pa_ALSa : CTTCCCTCCCGCGCCACCTGCCCGCCTCGCGCCGCGCC---CTCGCCGCC---ACCCGCATCAGGTGCTC : 145
Ps_ALS : ............................A.........---.........---................. : 241
Pa_ALSb : .CAG.......................G..........GCC.........GCC................. : 160
Pi_ALS : .CA...........G...........CG..........GCC.........GCC................. : 279

 * 300 * 320 * 340 *
Pa_ALSa : CACGGTGTCC---CCTTCGCCCGCCCCTCCCGCCACCGCGCTCCGCCCATGGGGCCCCACCGAGCCCCGC : 212
Ps_ALS : ..........---......................................................... : 308
Pa_ALSb : ..........TCC................................G..G..................... : 230
Pi_ALS : ..........TCC...........G....................G..G.........G........... : 349

 360 * 380 * 400 * 420
Pa_ALSa : AAGGGCGCCGACATCCTCGTCGAGGCCCTGGAGCGCTGCGGCATCAGCGACGTCTTCGCCTACCCCGGCG : 282
Ps_ALS : ...................................................................... : 378
Pa_ALSb : ...................................................................... : 300
Pi_ALS : .................................................................G.... : 419

 * 440 * 460 * 480 *
Pa_ALSa : GCGCCTCGATGGAGATCCACCAGGCGCTCACGCGCTCGCCGGCCATCACCAACCACCTCTTCCGGCACGA : 352
Ps_ALS : ...................................................................... : 448
Pa_ALSb : .......C................................C............................. : 370
Pi_ALS : .......C................................T............................. : 489

 500 * 520 * 540 * 560
Pa_ALSa : GCAGGGGGAGGCGTTCGCCGCGTCCGGGTACGCCCGCGCCTCCGGCCGCGTCGGGGTCTGCGTCGCCACC : 422
Ps_ALS : ...................................................................... : 518
Pa_ALSb : ..................G..C...........G.................................... : 440
Pi_ALS : ..................G..C...........G.................................... : 559

 * 580 * 600 * 620 *
Pa_ALSa : TCCGGCCCCGGCGCCACCAACCTCGTCTCCGCGCTCGCCGACGCTCTGCTCGACTCCATCCCGATGGTCG : 492
Ps_ALS : .................T.................................................... : 588
Pa_ALSb : ............................................C.................C....... : 510
Pi_ALS : ............................................C.................C....... : 629

 640 * 660 * 680 * 700
Pa_ALSa : CCATCACGGGGCAGGTCCCGCGCCGCATGATCGGCACGGACGCCTTCCAGGAGACGCCGATCGTGGAGGT : 562
Ps_ALS : .............................................................T........ : 658
Pa_ALSb : ..........C..........................C....................C..T........ : 580
Pi_ALS : ..........C..........................C....................C..T..C..... : 699

 * 720 * 740 * 760 *
Pa_ALSa : CACCCGTTCCATCACCAAGCACAATTACCTGGTCCTTGACGTGGAGGACATCCCCCGCGTCATTCAGGAA : 632
Ps_ALS : ...................................................................... : 728
Pa_ALSb : ........................C...........C.....C....................C...... : 650
Pi_ALS : ........................C.....C...........C....................C...... : 769

 780 * 800 * 820 * 840
Pa_ALSa : GCCTTCTTCCTCGCCTCCTCCGGCCGGCCGGGGCCGGTGCTGGTCGACATCCCCAAGGACATCCAGCAGC : 702
Ps_ALS : ...................................................................... : 798
Pa_ALSb : ................................C..................................... : 720
Pi_ALS : ................................C..................................... : 839

 * 860 * 880 * 900 *
Pa_ALSa : AGATGGCCGTGCCTGTCTGGGACGCGCCAATGAGTCTGCCAGGGTACATTGCTCGCCTCCCTAAGCCGCC : 772
Ps_ALS : ...................................................................... : 868
Pa_ALSb : .......T.....C..............T......................................... : 790
Pi_ALS : .......T.....C..............T......................................... : 909

 920 * 940 * 960 * 980
Pa_ALSa : GGCTACCGAATTGCTTGAGCAGGTCCTGCGTCTGGTTGGTGAGGCTCGGCGCCCAATTCTGTATGTTGGT : 842
Ps_ALS : ...................................................................... : 938
Pa_ALSb : ...........................................T..........G............... : 860
Pi_ALS : ...............C...........................T..........G............... : 979

 * 1000 * 1020 * 1040 *
Pa_ALSa : GGTGGCTGCTCTGCGTCCGGCGAGGAGTTGCGCCGCTTTGTTGAGCTCACTGGGATCCCAGTGACAACTA : 912
Ps_ALS : ...................................................................... : 1008
Pa_ALSb : ......A....................C.......................................... : 930
Pi_ALS : ...........................C.......................................... : 1049

 1060 * 1080 * 1100 * 1120
Pa_ALSa : CCCTCATGGGTCTTGGCAACTTCCCCAGCGATGACCCACTGTCTCTGCGTATGCTTGGGATGCATGGTAC : 982
Ps_ALS : ...................................................................... : 1078
Pa_ALSb : .....................................G................................ : 1000
Pi_ALS : .....................................G..........................C..... : 1119

 * 1140 * 1160 * 1180 *
Pa_ALSa : AGTCTACGCCAATTACGCGGTAGATAAGGCTGACCTGCTGCTTGCATTTGGTGTGCGGTTTGATGACCGT : 1052
Ps_ALS : ...................................................................... : 1148
Pa_ALSb : .........G........C..................................................C : 1070
Pi_ALS : ..................C..................................................C : 1189

 1200 * 1220 * 1240 * 1260
Pa_ALSa : GTGACTGGAAAAATAGAGGCTTTTGCAAGCAGGTCCAAGATTGTGCACATTGACATTGATCCAGCTGAGA : 1122
Ps_ALS : ...................................................................... : 1218
Pa_ALSb : ...........G.......................................................... : 1140
Pi_ALS : ...........G.......................................................... : 1259

 * 1280 * 1300 * 1320 *
Pa_ALSa : TTGGCAAGAACAAGCAGCCACACGTCTCCATTTGTGCAGATGTCAAGATCGCTTTGGAGGGCTTGAATTC : 1192
Ps_ALS : ...................C.................................................. : 1288
Pa_ALSb : ...................................................................C.. : 1210
Pi_ALS : ...................................................................C.. : 1329

 1340 * 1360 * 1380 * 1400
Pa_ALSa : TCTTCTGCTAAATGGGAGCAAAACACACAAGAGTTTAGATTTTAGTTCGTGGCATGAGGAGTTGGACCAG : 1262
Ps_ALS : ...................................................................... : 1358
Pa_ALSb : ...C.........................................C........................ : 1280
Pi_ALS : .............................................C........................ : 1399

 * 1420 * 1440 * 1460 *
Pa_ALSa : CAGAAGAGGGAGTTTCCTCTGGGATTCAAAACTTTTGGTGAGGCGATCCCACCACAATATGCTATCCAGG : 1332
Ps_ALS : ...................................................................... : 1428
Pa_ALSb : ....................A................................................. : 1350
Pi_ALS : ...................................................................... : 1469

 1480 * 1500 * 1520 * 1540
Pa_ALSa : TACTGGATGAGCTGACCAAAGGGGAGGCGATCATTGCCACTGGTGTTGGGCAGCACCAGATGTGGGCGGC : 1402
Ps_ALS : ...................................................................... : 1498
Pa_ALSb : ...................................................................A.. : 1420
Pi_ALS : ...................................................................A.. : 1539

 * 1560 * 1580 * 1600 *
Pa_ALSa : TCAGTATTACACGTACAAGCGGCCACGTCAGTGGCTGTCTTCGGCTGGTCTTGGAGCAATGGGGTTTGGG : 1472
Ps_ALS : ...................................................................... : 1568
Pa_ALSb : ............T..............G.......................G.................. : 1490
Pi_ALS : ............T..............G.......................G.................. : 1609

 1620 * 1640 * 1660 * 1680
Pa_ALSa : TTGCCAGCTGCAGCTGGTGCTGCTGTGGCCAACCCAGGTGTCACAGTTGTTGACATTGATGGAGATGGTA : 1542
Ps_ALS : ...................................................................... : 1638
Pa_ALSb : ...................................................................... : 1560
Pi_ALS : ...................................................................... : 1679

 * 1700 * 1720 * 1740 *
Pa_ALSa : GCTTCCTCATGAATATTCAGGAGTTGGCACTGATTCGTATTGAGAACCTCCCTGTTAAGGTGATGATACT : 1612
Ps_ALS : ...................................................................... : 1708
Pa_ALSb : .....................................C..C...........G................. : 1630
Pi_ALS : .....................................C..C...........G................. : 1749

 1760 * 1780 * 1800 * 1820
Pa_ALSa : GAACAACCAACATCTGGGAATGGTGGTGCAGTGGGAGGACAGGTTTTACAAGGCCAATCGGGCGCACACT : 1682
Ps_ALS : ...................................................................... : 1778
Pa_ALSb : .....................................................................G : 1700
Pi_ALS : .....................................................................G : 1819

 * 1840 * 1860 * 1880 *
Pa_ALSa : TACCTTGGGAACCCAGAAAATGAGAGTGAGATATATCCAGATTTTGTGACGATTGCCAAGGGGTTCAATG : 1752
Ps_ALS : ...................................................................... : 1848
Pa_ALSb : ........................................................T..A.......... : 1770
Pi_ALS : .....G..................................................T..A.......... : 1889

 1900 * 1920 * 1940 * 1960
Pa_ALSa : TTCCTGCTGTTCGTGTGACAAAGAAGAGTGAAGTCCGTGCAGCAATCAAGAAGATGCTTGAGACTCCAGG : 1822
Ps_ALS : ...................................................................... : 1918
Pa_ALSb : .......A...........................................CA................. : 1840
Pi_ALS : .......A.................A.........................C.................. : 1959

 * 1980 * 2000 * 2020 *
Pa_ALSa : GCCATACTTGTTGGATATCATCGTCCCTCACCAGGAGCATGTGCTGCCTATGATCCCCAGCGGTGGTGCT : 1892
Ps_ALS : ...................................................................... : 1988
Pa_ALSb : .......................................C.............................. : 1910
Pi_ALS : .......................................C.............................. : 2029

 2040 * 2060 * 2080 * 2100
Pa_ALSa : TTCAAGGACATTATCATGGATGGTGATGGTAGGATTGCTTATTAATCAAAACTTCGGCAAGAGCTCGACC : 1962
Ps_ALS : ...................................................................... : 2058
Pa_ALSb : ....................G..C.....C.................G......T........------- : 1973
Pi_ALS : ....................G..C.....C.................G...............------- : 2092

 * 2120 * 2140 * 2160 *
Pa_ALSa : TATATGACCTAGAGGTGCAGTGTGCGTGATCAGCATGATGCTGGTGTATGATGTATCAACTACTAAGGTT : 2032
Ps_ALS : ...................................................................... : 2128
Pa_ALSb : ............G......A.....C............................................ : 2043
Pi_ALS : ...................A.....C............................................ : 2162

 2180 * 2200 * 2220 * 2240
Pa_ALSa : GTGAAGTGTGTCAACTGTGAACCATGTATTTCGCTAGTTTGCTTGTTTGATGAGCCTGGTATGGTAATCT : 2102
Ps_ALS : ...................................................................... : 2198
Pa_ALSb : ...............................................C............T......... : 2113
Pi_ALS : ...............................................C............T......... : 2232

 * 2260 * 2280 * 2300 *
Pa_ALSa : TACCTAGCTCTGAACCTTAAAATAAAAGCGCAT------------------------------------- : 2135
Ps_ALS : ..............................TT.TTCCTAATTTA-CAGCTAGTTGTTTACTATCTCAGAA : 2267
Pa_ALSb : .....G............G..........A.T.TTCTTTCAAAAAAAAAAAAA----------------- : 2166
Pi_ALS : .....G............G..........A.T.TTCTTTCTATCTCAGCTACTCTGGTTTTGTAGTCGTT : 2302

 2320 * 2340
Pa_ALSa : ------------------------------------- : -
Ps_ALS : AAAAAA------------------------------- : 2273
Pa_ALSb : ------------------------------------- : -
Pi_ALS : TGATTTGCTCCGAGTAAATGTTTTTCTGTGTCCGGAT : 2339
